# Changes in the Honeybee Antioxidant System after 12 h of Exposure to Electromagnetic Field Frequency of 50 Hz and Variable Intensity

**DOI:** 10.3390/insects11100713

**Published:** 2020-10-18

**Authors:** Paweł Migdał, Agnieszka Murawska, Aneta Strachecka, Paweł Bieńkowski, Adam Roman

**Affiliations:** 1 Department of Environment Hygiene and Animal Welfare, Wroclaw University of Environmental and Life Sciences, 25 C.K. Norwida st., 51-630 Wroclaw, Poland; agnieszka.murawska@upwr.edu.pl (A.M.); adam.roman@upwr.edu.pl (A.R.); 2 Institute of Biological Basis of Animal Production, Faculty of Biology, Animal Sciences and Bioeconomy, University of Life Sciences in Lublin, Akademicka 13, 20-950 Lublin, Poland; aneta.strachecka@up.lublin.pl; 3 Telecommunications and Teleinformatics Department, Wroclaw University of Science and Technology, 27 Wybrzeze Wyspianskiego st., 50-370 Wroclaw, Poland; pawel.bienkowski@pwr.edu.pl

**Keywords:** honeybee, antioxidant system, E-field, 50 Hz, superoxide dismutase (SOD), catalase (CAT), total antioxidant potential (FRAP)

## Abstract

**Simple Summary:**

The honeybee is one of the most important links in the food production chain. In the environment of bee life, there are many threats that they have to face. Among them, we can distinguish pesticides, climate change, and predators. The intensive development of technology resulted in filling the natural environment with E-field of various frequencies and intensities. The study investigated the effect of the E-field with a frequency of 50 Hz at various intensities on the antioxidant system of the honeybee. The exposure of the bees lasted 12 h. The activity of the antioxidant system was investigated in hemolymph collected from young bees. Research has been undertaken in this direction because research by other authors has taken years to stimulate this system through the E-field. The superoxide dismutase (SOD), Catalase (CAT), and total antioxidant potential (FRAP) activity as major antioxidant enzymes were characterized. Research showed changes in the activity of SOD, CAT, and FRAP in all tested groups compared to the control group. The study of E-field appearing in the life of a honeybee enables an understanding of the impact of this factor on its functioning because food production depends on the integrity of this pollinator.

**Abstract:**

In recent years, on a global scale, more and more reports of a phenomenon called CCD (Colony Collapse Disorder) have been reported. In addition to pesticides, diseases, and other environmental stressors, electromagnetic fields are also mentioned as one of the possible causes of CCD. One of the body’s first lines of defense against harmful factors is the antioxidant system. We hypothesized that electromagnetic field upregulate the activity of SOD (superoxide dismutase), CAT (catalases), and changed FRAP (total antioxidant potential) in honeybee hemolymph. In our research, 12 h bee’s exposure to E-field was analyzed to determine changes in the antioxidant system. The frequency of 50 Hz and various intensities were used: 5.0 kV/m, 11.5 kV/m, 23.0 kV/m, and 34.5 kV/m. Superoxide dismutase was characterized by four times higher activity in the study groups as compared to the control group. Catalase activity in all groups was characterized by statistically significantly different activity between the groups. The highest activity was recorded in the 34.5 kV/m group. The lowest activity was recorded in the 11.5 kV/m group. A relationship was found between different E-field intensities and changes in the antioxidant system.

## 1. Introduction

As one of the key pollinators, the honeybee is exposed to many different threats in the environment, which can cause changes in its organism. The pollination of entomophilic plants depends on the proper functioning of these insects in the environment. It is estimated that 75–95% of plants species require pollinator support to yield or increase yield [1]. They contribute to the pollination of over 180,000 different plant species, including arable crops [2]. According to Gallai et al. [3], the activity of pollination in terms of the global economy can be as high as $217 billion.

Unfortunately, in recent years, on a global scale, more and more reports of a phenomenon called CCD (Colony Collapse Disorder) have been reported. It is characterized by the disappearance of entire colonies or the abandonment of their nests (hives) with only brood, food supplies, and a queen with a small number of bees [4]. So far, no unequivocal diagnosis of the cause of this phenomenon has been made. Many different factors are suspected: new disease entities, pests, inadequate hive hygiene, next-generation pesticides, artificial fertilizers, breeding work leading to a biodiversity reduction, and an artificial electromagnetic field [5]. Technological devices have become indispensable elements of most people’s everyday life. Technology supports different areas of life, making it easier to do many things. It is estimated that over three billion people are exposed to the E-field every day [6]. All electrical devices are emitters of the electromagnetic field (EMF), which fills the natural environment more and more tightly. The power line frequency in Europe, Australia, some parts of South America, and in the east of Japan is 50 Hz (in the rest of the world 60 Hz) [7,8]. At a distance of a few meters from the 220 and 400 kV/m transmission lines and at a height of 2 m above the ground, electromagnetic field strength has most often the values of 10–12 kV/m (at the frequency of 50 Hz). Further, at a distance over 20 m from the extreme conductors of the transmission lines (also measured 2 m above the ground), these values are slightly lower, i.e., 5–7 kV/m [8,9,10]. If worker honeybee flies at a high of about 2 m in an open, unobstructed space near the power line is exposed to an electromagnetic field with an intensity of 10–12 kV/m. If a tree, building, or other obstacles appears on its way, the bee flies about five and more meters above the ground (E-field intensity 5–7 kV/m). As a result, when approaching the transmission lines, the bee is exposed to intensities over 12 kV/m.

The studies of other authors indicate the multidirectional influence of the electromagnetic field of various frequencies on living organisms. Many studies show a relationship between low-frequency electromagnetic fields and cell proliferation, adherence, and differentiation in in-vitro cultures [11,12]. Bone marrow cells changed their structure at a frequency of 15–30 Hz. According to some scientists, changes can occur at the biochemical and genetic level [13,14]. Experiments on birds with the use of magnets disturbing the magnetic field showed that they lost their orientation in space [15,16]. It has also been proven that birds avoid places with increased electromagnetic background, especially those with high frequencies [17]. The frog *Xenopus laevis* was used in research on the influence of electromagnetic fields with a frequency of 50 Hz and an intensity of 50.76 A m^−1^ to 60.69 A m^−1^ on development indicators [18]. It was observed that the activity of these fields accelerated the mean metamorphosis time of tadpoles by 2.4 days. Regarding fish, the influence of the 1 Hz field on the concentration of melatonin and cortisol in *Salvelinus fontinalis* was investigated. The obtained results indicated a disturbance in the circadian rhythm of the fish [19]. In the case of the Nematoda, radio waves (50 MHz to 1 GHz) with prolonged exposure caused thermal shock, acceleration of puberty by 40%, and an increase in the concentration of stress hormones compared to the control group [16,20,21]. The grape snail subjected to the influence of the electromagnetic field of various frequencies, from about 8 Hz to 300 Hz, at different exposure times (from 0.5 h to two months), showed changes in nerve cells, linear increase in mortality, damage to lysosomal membranes [22,23]. Radiofrequency EMFs can cause various chemical effects, including the degradation of large molecules in cells and disrupting homeostasis [24].

During biological reactions, oxygen molecules can turn into hazardous by-products called reactive oxygen species (ROS) [25]. These reactive oxygen species can damage cellular components such as proteins, lipids, and DNA [26]. The free radical formation can occur in a variety of ways, including ultraviolet light, drugs, lipid oxidation, immune responses, radiation, stress, smoking, alcohol, and biochemical redox reactions [27,28]. It is known that exposure to EMF increases free radical concentration and traceability and may affect the recombination of radical pairs. The overproduction of ROS as a result of external factors causes oxidative stress [29]. Oxidative stress is a state where the antioxidant defense system is unable to prevent the harmful effects of free radicals [30]. This physiological state upregulates the activity of enzymes such as superoxide dismutase (SOD) and catalases (CAT) to protect an organism from damage caused by ROS. SOD catalyzes the partitioning of superoxide radicals to hydrogen peroxide (H_2_O_2_) and further CAT catalyzes H_2_O_2_ decomposition to water (H_2_O) and molecular oxygen (O_2_) [31,32]. Superoxide dismutase and catalases belong to the class of oxidoreductases and are involved in the inactivation of the superoxide radical. Both of these enzymes belong to the basic enzymes that make up the antioxidant triad. The life span of the organism depends to some extent on the efficiency of the anitoxidant system (the better the neutralization of ROS, the longer the organism can live) [33]. This system is also present in insects, including the honeybee. It is not as efficient as in other insects’ organisms and has limited protection against reactive oxygen species. This is due to a small number of genes encoding both enzymatic and non-enzymatic antioxidant proteins [34,35]. Analysis of SOD and CAT activity gives information about organism oxidative stress. The antioxidant power of the hemolymph can be assessed by ferric ion reducing antioxidant power (FRAP) assay [36]. FRAP analysis gave precise information about the general stimulation of the antioxidant system. 

The higher level of ROS the higher probability of organism self-oxidation, so it was necessary that the organism create an antioxidant system to protect its cells. If the 50 Hz electromagnetic field deregulates the antioxidant system, it causes disturbance of disproportionation (by SOD and CAT) of hydrogen peroxide to create two different oxygen-radical species in the honeybee exposed to this stress factor. Disturbance of mentioned reactions is caused among others by pesticides which are classified as one of the CCD factors [37]. Thus, we suppose that EMF can be one of the causes of CCD.

We hypothesized that electromagnetic field upregulate the activity of SOD, CAT, and changed FRAP in honeybee hemolymph.

Our study aimed to show the effect of the electromagnetic field at 50 Hz and variable intensity of 5, 11.5, 23, and 34.5 kV/m on honeybee antioxidant systems parameter such as SOD, CAT, and FRAP after 12 h of exposition.

## 2. Material and Methods

### 2.1. Research Material

Ten honeybee (*Apis mellifera carnica*) colonies from Apiary at Research and Didactic Station in Swojczyce, Wroclaw, Poland were treated against *Varroa destructor* (amitraz fumigation four times at 4-day intervals, a 12.5 mg/tablet Amitraz^®^ Biowet Pulawy), before the experiment. To monitor the number of *Nosema* spp. spores we used the hemocytometer method (30 bees per hive in three repetitions). On the 20th day of apian development, the combs with the already sealed worker brood were transferred to the laboratory and placed in an incubator with a controlled temperature of 34.4 ± 0.5 °C and relative humidity of 70% ± 5%. In incubator honeybees workers had ensured honey and bee bread ad libitum. One-day-old honeybee workers were placed in wooden cages (200 × 150 × 70 mm) each containing 100 workers and two inner feeders with sucrose solution at a concentration of 1 mol/dm^3^ ad libitum. Each group (experimental and control) consisted of 10 cages. The research material consisted of the two-days old of honeybee workers. The method according to Migdał et al. [38].

### 2.2. Hemolymph Analyses

Hemolymph was taken from 100 alive honeybees worker from each group after exposure to E-field, by removing the antennae with sterile tweezers. Hemolymph was conserved in 20 µL glass capillary [39]. The test tubes were placed on the cooling block during operation. The prepared tubes were transferred to a cryo-box and then frozen at −80 °C [40]. Superoxide dismutase (SOD) activities were determined using a commercial Sigma-Aldrich 19160 SOD determination kit. Catalase activities (CAT) were determined using a commercial kit from EnzyChromTMCatalase Assay Kit (ECAT-100). The antioxidant capacity of the hemolymph was determined by FRAP (Ferric ion reducing antioxidant power), the determination of this parameter was carried out according to a procedure developed by Benzie and Strain [36] and subsequently modified by Thaipong et al. [41].

### 2.3. Exposure to the Electromagnetic Field

The experiment was carried out from 15 to 30 July 2019. Bees in the experimental groups were exposed to the following 50 Hz EMF intensity 5.0 kV/m, 11.5 kV/m, 23.0 kV/m, and 34.5 kV/m for 12 h starting at 6 am. Two first intensities are common in the environment and honeybee can be exposed to them during foraging. The other two were used to investigate whether higher values will cause different changes in the antioxidant system. The control group was not treated to the artificial electromagnetic field, they were under the influence of measured electromagnetic field <2.0 kV/m. The group’s name is EMF intensity. Exposure time was 12 h for all groups. The control group was marked with the letter C. The 50 Hz E-field was generated in the exposure system in the form of a plate capacitor like in a method described by Migdał et.al. [38]. All groups (experimental and control) during the whole experiment were keeping at the same controlled temperature (25 ± 0.5 °C). Changes in homogeneity and stability of the EMF intensity in the emitter were lower than ± 5%. The intensity and the homogeneity of the EMF in the test and control area were measured by an LWiMP accredited testing laboratory (certification AB-361 of Polish Centre for Accreditation) using an ESM-100-m No. 972153 with calibration certificate LWiMP/W/070/2017 dated on 15 February 2017 issued by the accredited calibration laboratory PCA AP-078. 

### 2.4. Data Evaluation

The normality of the data distribution was analyzed by the Shapiro-Wilk test. The statistical significance of the difference in the mean values of data between groups was determined by the Kruskal–Wallis test. The statistical significance level of α = 0.05 was used. RStudio was used to conduct all tests [42].

## 3. Results

### 3.1. Superoxide Dismutase Activity (SOD)

**Figure 1:**
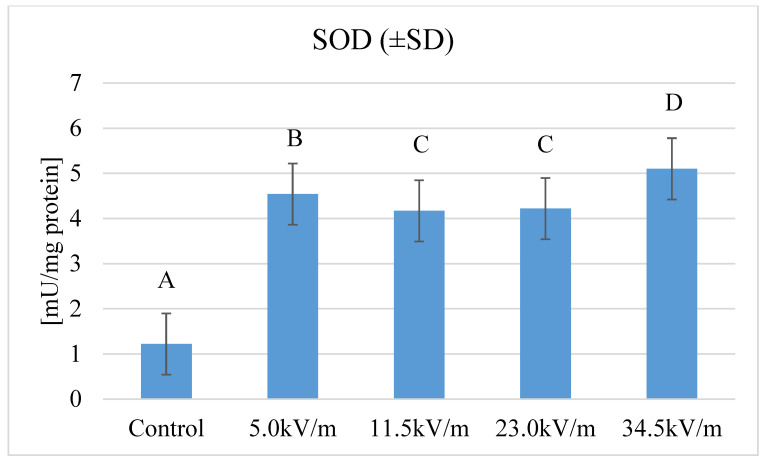
Superoxide dismutase (SOD) activity in honeybee hemolymph after exposure to the E-field (50 Hz and different intensities). The group name is the f E-field intensity. The control group was marked with the letter C. The control groups were not treated to the artificial electromagnetic field, they were under the influence of an electromagnetic field lower than 2.00 kV/m. Statistical differences between the mean values for groups are marked with different letters. Different letters mean the difference is at the *p*-value ≤ 0.05 significance level.

Superoxide dismutase was characterized by four times higher activity in the study groups as compared to the control group (Figure 1). In all studied groups, statistically, significantly higher SOD activity was observed than in the control group (*p*-value < 2.2 × 10^−16^). The highest activity was recorded in the group with the highest E-field intensity. The lowest activity was recorded in the control group. The differences between the lowest and highest activity were statistically significant. Regarding intensities of 11.5 kV/m and 23.0 kV/m, no statistically significant differences were observed between these groups.

### 3.2. Catalase Activity (CAT)

**Figure 2:**
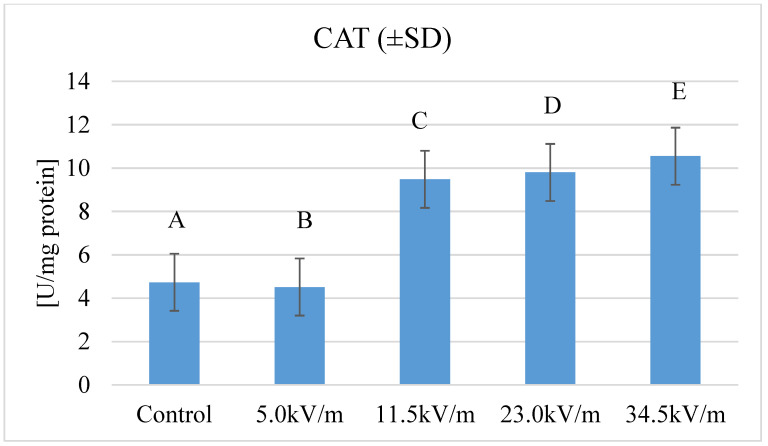
Catalase (CAT) activities in honeybee hemolymph after exposure to the E-field (50 Hz and different intensities). Details as in Figure 1. Statistical differences between the mean values for groups are marked with different letters. Different letters mean the difference is at the *p*-value ≤ 0.05 significance level.

Catalase activity in all groups was characterized by statistically significantly different activities between the groups (*p*-value < 2.2 × 10^−16^) (Figure 2). The highest activity was observed for the group with the highest E-field intensity (34.5 kV/m). The lowest activity was recorded for the group with the lowest E-field intensity (5.0 kV/m). It was an activity lower than the catalase activity in the control group. The biggest difference in catalase activity was noted between the 5.0 kV/m group and the 34.5 kV/m group.

### 3.3. Total Antioxidant Potential (FRAP)

**Figure 3:**
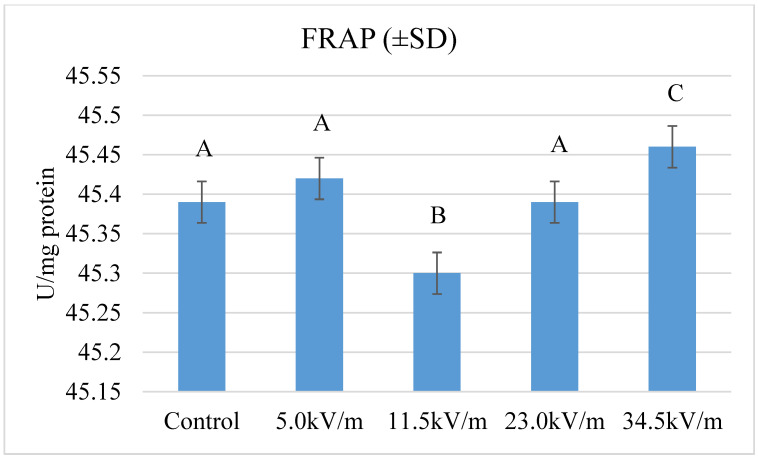
Ferric reducing antioxidant power (FRAP) in honeybee hemolymph after exposure to the E-field (50 Hz and different intensities). Details as in Figure 1. Statistical differences between the mean values for groups are marked with different letters. Different letters mean the difference is at the *p*-value ≤ 0.05 significance level.

Total antioxidant potential showed statistically significant differences between the groups (Figure 3) (*p*-value < 1.664 × 10^−13^). The highest activity was recorded in the 34.5 kV/m group. The lowest activity was recorded in the 11.5 kV/m group. The difference between these groups was statistically significant. There were no statistically significant differences between the control groups, 5.0 kV/m, and 23.0 kV/m. The 11.5 kV/m and 23.0 kV/m groups were characterized by lower activity than the control group, in the other groups, the activity was higher than in the control group.

## 4. Discussion

### 4.1. Superoxide Dismutase Activity (SOD)

Our research showed that 12-h exposure of honeybee workers to selected E-field intensities causes a statistically significant increase in the activity of superoxide dismutase (Figure 1). This enzyme is responsible for the catalysis of the reaction of the superoxide radical (O_2_^−^) splitting into molecular oxygen (O_2_) and hydrogen peroxide (H_2_O_2_). Superoxide is a by-product, causing much cellular damage if not controlled [43]. At the same time, this system is one of the first lines of defense against pathogens and environmental stressors [31,32]. Our research revealed four times higher SOD activity than in the studies performed by Strachecka et al. [44], which investigated the influence of coenzyme Q10 on SOD for bees aged two-days. This proves that the antioxidant system was highly stimulated under the influence of E-field. Much lower SOD activity was obtained by Collins et al. [45] investigating the stored semen in queen bees. Li et al. [46], when examining the effect of pollen nutrition, showed SOD activity above 2.6 U/mg in the middle intestine. Higher results were obtained by Nikolića et al. [47] who analyzed homogenates of whole bees from areas with varying degrees of anthropopressure. Comparing SOD activity in bees from urban, industrial, and control areas, showed that bees from the polluted area have increased response of the antioxidative enzymes. Differences between our and those mentioned above results may be related to different bee tissues collected for research. Despite the different activities, all the studies confirm the significant influence of external factors on the activity of SOD. These environmental stressors are classified as one of the CCD cause-factors [33,37]. In our studies, the 50 Hz electromagnetic field deregulates the activity of SOD and CAT which are responsible for the disproportionation of hydrogen peroxide to create two different oxygen-radical species in the honeybee. We can suppose that the electromagnetic field impacts the honeybee antioxidant system like other stress factors (pesticides or chemicals), the activity of which is related to the occurrence of the CCD phenomenon. Therefore E-field could be classified as one of the CCD cause-factors, but this statement requires a larger number of studies. The influence of E-field from power line frequency to radiofrequency on SOD activity has been confirmed in relation to many living organisms and their tissues [24,38,48]. In the studies of Strachecka et al. [37], two-days old bees have more than twice as high SOD activity than bees in our experimental groups. In previous research, we observed the increase in SOD activity under the influence of the 50 Hz electromagnetic field with shorter exposure time [38]. After 12 h of exposure to E-field, the similar activity of SOD was recorded in previous research for 5.0 kV/m at three and six hours exposure and for the 34.5 kV/m group at one and six hours exposure. The increase in SOD activity is also observed at different times of the season. Korayem et al. [49] research showed that the higher the seasonal activity of workers, the higher the SOD activity. According to Orčić et al. [50], high radical production is associated with increased metabolic processes resulting from increased activity, i.e., the oxygen demand of tissues during the flight.

### 4.2. Catalase Activity (CAT)

The activity of the oxidoreductive catalase is one of the most important mechanisms of protection of the body against oxidative damage caused by reactive oxygen species (ROS). Additionally, catalase is one of the most active enzymes capable of breaking down millions of hydrogen peroxide particles into oxygen and water every second [51]. Catalase contributes to the protection of the organism against the negative influence of external factors, e.g., the studies by Mockett et al. [52] showed that with increased activity of catalase, fruit flies were characterized by higher resistance to stress caused by high temperature. Our own research confirms the influence of E-field on catalase activity at 12 h exposure (Figure 2). In the studies of Strachecka et al. [37], two days old bees have similar CAT activity to bees from our control group and 5.0 kV/m, while bees in other experimental groups have higher CAT activity. Catalase activity determined in the authors’ own research in the study groups was different than in the studies by Sagona et al. [53] who studied changes in catalase activity in age polyethism. Newly emerged bees in this study have catalase activity from 7.01 mU/mg (thorax), through 30.51 mU/mg (verticuli), to 159.51 mU/mg (wings). Differences between our and Sagona et al. [53] studies are caused by different research material. Catalase activity in worker bee hemolymph in the studies of Weirich et al. [54] was around 0.25 mU/ug, which is a lower value than received in our own studies. Similar catalase activity was observed in the work with shorter exposure times and the same E-field parameters. It was also found that extending the exposure time of honeybee workers to E-field up to 12 h causes a further increase in the activity of this enzyme, this does not apply to the 5.0 kV/m group, which was characterized by similar activity to the previous work [38]. Our own research confirms that 12 h exposure to the E-field with a frequency of 50 Hz significantly affects the activity of catalase. Similar fluctuations in catalase activity were observed in the studies of Nikolića et al. [47] who investigated the effect of sublethal concentrations of Cu, Pb, and Cd on catalase activity under laboratory conditions.

### 4.3. Total Antioxidant Potential (FRAP)

The authors’ research showed statistically significant differences in the total antioxidant potential in the 11.5 kV/m and 34.5 kV/m groups. The remaining groups did not differ significantly from the results obtained in the control group (Figure 3). We obtained similar results in the previous work with shorter exposure times [38]. Extending the time did not result in significant changes to FRAP. A similar activity for FRAP in bees at the age of two days was obtained by Strachecka et al. [37] (about 47 µmol Fe^2+^ L^−1^) who investigated the effects of bromfenvinphos in the treatment of bees against varroosis. Bees over seven days old have the FRAP activity much lower than those obtained in our own research (about 34 umol Fe^2+^ L^−1^). In another work by Strachecka et al. [55] FRAP changed with changes in *Osmia rufa* activity (from about 32 to 48 umol/L). This may indicate an increased supply of free radicals that must be neutralized. Our own research has shown that the intensity of 34.5 kV/m contributes to the increased activity of FRAP (45.47 U/mg), which may indicate the influence of E-field on the proteolytic system. In order to accurately determine the effects of E-field on the proteolytic system of worker honeybees, levels of non-enzymatic antioxidants would need to be determined. 

## 5. Conclusions

Technology is becoming a challenge for many groups of animals, including bees. Especially, this new factor dynamically changes in the range of frequencies and intensities that are used. This contributes to the ever-expanding E-field study. The results from this study provide basic data for future research regarding the influence of the electromagnetic field with a frequency of 50 Hz on the antioxidant system of the honeybee and will be an important step towards a comprehensive risk assessment of the environmental stressors on honeybees.
